# Hepatic Transcriptome Comparative In Silico Analysis Reveals Similar Pathways and Targets Altered by Legacy and Alternative Per- and Polyfluoroalkyl Substances in Mice

**DOI:** 10.3390/toxics11120963

**Published:** 2023-11-28

**Authors:** Dakota R. Robarts, Jiayin Dai, Christopher Lau, Udayan Apte, J. Christopher Corton

**Affiliations:** 1 Department of Pharmacology, Toxicology and Therapeutics, University of Kansas Medical Center, Kansas City, KS 66160, USA; 2 Center for Computational Toxicology and Exposure, US Environmental Protection Agency, Research Triangle Park, NC 27711, USA; 3 State Environmental Protection Key Laboratory of Environmental Health Impact Assessment of Emerging Contaminants, School of Environmental Sciences and Engineering, Shanghai Jiao Tong University, Shanghai 200240, China; 4 Center for Public Health and Environmental Assessment, US Environmental Protection Agency, Research Triangle Park, NC 27711, USA

**Keywords:** PFAS, toxicogenomics, PPARα, CAR, PXR, STAT5B, SREBP, GenX

## Abstract

Per- and poly-fluoroalkyl substances (PFAS) are a large class of fluorinated carbon chains that include legacy PFAS, such as perfluorooctane sulfonate (PFOS), perfluorooctanoic acid (PFOA), perfluorononanoic acid (PFNA), and perfluorohexane sulfonate (PFHxS). These compounds induce adverse health effects, including hepatotoxicity. Potential alternatives to the legacy PFAS (HFPO-DA (GenX), HFPO4, HFPO-TA, F-53B, 6:2 FTSA, and 6:2 FTCA), as well as a byproduct of PFAS manufacturing (Nafion BP2), are increasingly being found in the environment. The potential hazards of these new alternatives are less well known. To better understand the diversity of molecular targets of the PFAS, we performed a comparative toxicogenomics analysis of the gene expression changes in the livers of mice exposed to these PFAS, and compared these to five activators of PPARα, a common target of many PFAS. Using hierarchical clustering, pathway analysis, and predictive biomarkers, we found that most of the alternative PFAS modulate molecular targets that overlap with legacy PFAS. Only three of the 11 PFAS tested did not appreciably activate PPARα (Nafion BP2, 6:2 FTSA, and 6:2 FTCA). Predictive biomarkers showed that most PFAS (PFHxS, PFOA, PFOS, PFNA, HFPO-TA, F-53B, HFPO4, Nafion BP2) activated CAR. PFNA, PFHxS, PFOA, PFOS, HFPO4, HFPO-TA, F-53B, Nafion BP2, and 6:2 FTSA suppressed STAT5b, activated NRF2, and activated SREBP. There was no apparent relationship between the length of the carbon chain, type of head group, or number of ether linkages and the transcriptomic changes. This work highlights the similarities in molecular targets between the legacy and alternative PFAS.

## 1. Introduction

Per- and polyfluoroalkyl substances (PFAS) are a large class of over 12,000 synthetic chemicals (EPA CompTox Dashboard; https://comptox.epa.gov/dashboard/chemical-lists/PFASMASTER (accessed on 16 March 2022)). As these compounds are widely used in different scenarios, people are exposed to PFAS through a number of routes, leading to concerns about potential adverse health effects. In many cases, PFAS are structurally similar to fatty acids. The fluorination of these compounds provides extreme stability and leads to persistence in the environment and long half-lives in animals and humans. The combination of a fluorinated carbon chain of variable length and a polar functional head group in part determines toxicity [1]. Exposure to PFAS is associated with adverse health effects, including disruption of metabolic function, reduced kidney function, thyroid interference, cancer, immunosuppression, and hepatotoxicity [2,3,4]. The toxicity profiles of increasing concern, of the first set of widely used PFAS, such as perfluorooctane sulfonate (PFOS), perfluorooctanoic acid (PFOA), perfluorohexane sulfonate (PFHxS), and perfluorononanoic acid (PFNA) (called legacy PFAS), led to the creation and use in consumer products of many alternative PFAS. These were hypothesized to be less toxic compared to the legacy PFAS. While these alternative PFAS still possess a fluorinated carbon tail, the tails tend to be shorter and are linked to the head group, in many cases through one or more ether bonds, to help shorten the long half-life in mammals [5].

Compared to other PFAS, PFOS, PFOA, PFNA, and PFHxS are most frequently found in the environment and bioaccumulate in humans [6,7,8]. In humans, PFAS have been associated with liver injury and toxicant-associated fatty liver disease (TAFLD), a pathology similar to non-alcoholic fatty liver disease (NAFLD) [9,10,11]. In rodents, these compounds induce a number of effects in the liver, including lipid accumulation, increases in hepatocyte size, and liver weight [12]. Alternative PFAS, including ammonium perfluoro-2-methyl-3-oxahexanoate (HFPO-DA or GenX), perfluoro-(2,5,8-trimethyl-3,6,9-trioxadodecanoic)acid (HFPO4), perfluoro-2,5-dimethyl-3,6-dioxanonanoic acid (HFPO-TA), potassium 9-chlorohexadecafluoro-3-oxanonane-1-sulfonate (F-53B), 6:2 fluorotelomer sulfonic acid (6:2 FTSA), and 2-perfluorohexyl ethanoic acid (6:2 FTCA) and a by-product of PFAS production called perfluoro-2-([perfluoro-3-(perfluoroethoxy)-2-propanyl]oxy)ethanesulfonic acid (Nafion BP2), are compounds that are found in many cases to be accumulating in the environment and in the blood of people and animals [13,14,15,16,17]. A major issue regarding these emerging PFAS is that little is known about their adverse health effects, including their potential mechanisms of liver toxicity.

Most PFAS studied thus far are activators of peroxisome proliferator-activated receptor α (PPARα), a nuclear receptor family member expressed in the livers of rodents and humans. Chronic PPARα activation can lead to a number of toxicities, the most notable of which are increases in hepatocellular adenomas and carcinomas in mice, and increases in tumors in the liver, testis, and pancreas (known as the tumor triad) in rats [18,19]. Long(er)-term oral exposures to PFOA, PFOS, and HFPO-DA have been shown to cause increases in tumors of the liver, pancreas, and/or testis in rats [20,21]. In addition to PPARα, the legacy PFAS induced the activation of other transcription factors, including the constitutive activated receptor (CAR) and the pregnane X receptor (PXR) [22,23,24,25], the oxidant-induced Nrf2 [24,26,27], and the sterol regulatory element binding protein (SREBP), which regulates triglyceride and cholesterol synthesis [28,29,30]. To date, no comprehensive analysis of the transcript profiles induced by a large number of legacy and alternative PFAS have been compared to identify overlapping and unique molecular targets.

In the present study, ToxPrints, Attagene FACTORAL Assays, and the transcript profiles induced by 11 legacy and alternative PFAS were computationally compared to those transcript profiles of five known PPARα activators using pathway analysis and characterized gene expression biomarkers (Figure S1A). The biomarkers included those that are highly predictive of the modulation of six transcription factors important in lipid, metabolic, and cell growth homeostasis in the liver. We found that almost all of the PFAS examined activate PPARα, not surprising given legacy PFAS are well known to be activators of PPARα and the structural similarities between legacy and emerging PFAS. In addition, our analysis revealed other pathways that are commonly induced by most PFAS. This study illustrates that some alternative PFAS share liver molecular targets with the legacy PFAS, including PPARα-dependent and -independent pathways.

## 2. Materials and Methods

### 2.1. ToxPrint Clustering Analysis

ToxPrint was used to determine the chemical structure similarities across the compounds examined in this study. The binary ToxPrint structural features were downloaded from the US EPA CompTox dashboard (https://comptox.epa.gov/dashboard, (accessed on 1 June 2022)) and imported into RStudio (R Version 4.0.3; RStudio Team). Unsupervised hierarchical clustering was performed using the Euclidean distance method and ward.D2 clustering on the entire ToxPrint for each compound. This analysis was applied using the same methods, with the exception that the ToxPrint for the functional head group features for all PFAS ToxPrints were removed from the analysis before clustering.

### 2.2. Data Acquisition

Two data acquisition methods were used. First, some of the publicly available datasets were obtained from BaseSpace Correlation Engine (BSCE, https://basespace.illumina.com, 1 April 2022) which contains more than 20,000 genomic studies. Lists of differentially expressed genes (DEGs) were exported from BSCE. The lists were derived from comparisons between the chemical-treated and control-treated groups for the following chemicals: the PPARα activator compounds, including WY-14,643, fenofibrate, CP-865520, CP-775146, and CP-868388, and the PFAS PFNA, Nafion BP2, PFHxS, PFOA, and PFOS. The same statistical procedures were used to derive all of the DEGs in BSCE (*p*-value < 0.05 and ±1.2-fold change cutoff with no multiple test correction), and the methods used are described in detail in Kuperschmidt et al. (2010). Table 1 shows the respective PubMed identifier describing the studies from which the gene lists were derived [15,31,32,33,34,35,36,37,38] (Corton et al. submitted).

Second, not all of the alternative PFAS examined by RNA-Seq (HFPO-TA, HFPO-DA, HFPO4, F-53B, 6:2 FTSA, and 6:2 FTCA) were available in BSCE and were obtained from the authors of the original studies (Wang et al., 2017) (Upon Request). DEGs were derived using the Partek Flow Genomics Suite. Briefly, the count matrices were imported into the Partek Flow Server (Partek Inc., St. Louis, MO, USA) and the raw counts were normalized, and DEGs were calculated using the DESeq2 method, as previously described [39]. To increase the ability to compare responses across different gene expression platforms, the same statistical filters were used to identify the DEGs compared to their respective controls (*p*-value < 0.05 and ±1.2-fold change cutoff with no multiple test correction). Experimental designs for each dataset are found in Table S1.

### 2.3. BaseSpace Correlation Engine

The significantly altered gene lists from the HFPO-TA, HFPO-DA, HFPO4, F-53B, 6:2 FTCA, and 6:2 FTSA treatment groups were uploaded into BSCE and analyzed through their online interface (performed April 2022). Briefly, BSCE uses the ranked based comparison method, the Running Fisher Test, to determine the pair-wise correlation between any two gene lists (described in detail in [40]). From this online based software for each compound, all correlated pathways were exported, including the pathway nomenclature (database and pathway name), correlation *p*-value (converted to −log(*p*-value)), and correlation direction, and were then imported into RStudio (R Version 4.0.3; RStudio Team). If there was a negative correlation direction, the −log(*p*-value) was multiplied by −1 to indicate the correlation direction. The DEGs were correlated to the genes found in Gene Ontology (GO) and the Broad MSigDB canonical pathways. For each database list, if the total number of significantly altered pathways (using an |−log(*p*-value)| cutoff of 4) was >100, a filtering method was used to reduce the number of pathways to 100. To carry this out, for each individual pathway, the number of significant −log(*p*-value)s were counted across all compounds, excluding the PPARα activators. The total number of significant alterations was then ranked in descending order. The top 100 were then included in the heat map.

For the predictive biomarker analysis, each biomarker gene list predictive of STAT5, AhR, PPARα, CAR, SREBP, and NRF2 modulation was uploaded into BSCE. These DEG lists were then compared to the DEG lists for each treatment group using the Running Fisher Test. The correlation *p*-value and correlation direction of each predictive biomarker were exported from BSCE and imported into RStudio for heatmap construction using the same methods as in the BSCE database analysis. A |−log(*p*-value)| ≥ 4 was considered significant activation or suppression based on prior studies [22,38,41,42].

### 2.4. Principal Component Analysis

Principal Component Analysis (PCA) was used as a form of data reduction to visualize similarity across groups. The significantly changed DEG list (the DEG lists used in the BSCE analysis) for each compound was imported into RStudio (R Version 4.0.3; RStudio Team). DEG lists were used to perform the analysis on a fold-change scale utilizing the stats (Version 3.6.2) package. The data were scaled to the z-score, and then the PCA was calculated by a singular value decomposition of the centered and scaled data. After plotting (R package ggplot2, Version 3.35), successive subclusters were examined using the same method.

### 2.5. Ingenuity Pathway Analysis

Each DEG list for each compound (the same used in the BSCE analysis) was uploaded into the Qiagen Ingenuity Pathway Analysis (IPA) software (IPA, Ingenuity Systems, www.ingenuity.com). The analysis focused on canonical pathways and upstream regulators, and the findings were exported from the software. In the upstream regulator analysis, the transcriptional regulators and ligand binding nuclear receptors were examined as annotated by IPA, as previously described [43]. Heatmaps were then generated for these groups (canonical pathways, transcriptional regulators, and ligand binding nuclear receptors). If there were >100 total entities altered across all compounds, a filtering method was used to reduce the number to 100 total pathways. For this method, a counting approach (similar to the BSCE method above) was used to rank the pathways. A significantly altered pathway was defined as a pathway that had a *p*-value < 0.05. The significantly altered pathways were then tallied across all PFAS treatment groups for each pathway. This count was then ordered in a descending fashion, and the top 100 pathways were used to build a heatmap. The PPARα activators were excluded from the count, as the goal was to examine only the similarities of the PFAS alterations. This analysis was performed in May 2022.

### 2.6. FACTORIAL Activation Assay

The FACTORIAL activation assay table was downloaded from the supplementary data section of the Houck et al., 2021 study [44]. These data were then uploaded into R Studio (R Version 4.0.3; RStudio Team). The data were first filtered for compounds that overlapped with those described in our study. Then, all activity assays that elicited no effect across all the PFAS of interest were removed. The remaining activity assays were used in the clustering analysis described below. The potency values (AC_50__Modified) and type of assay (CIS vs. TRANS) features were retained for the heatmap construction.

### 2.7. Heatmap Construction

Heatmaps were constructed using the R packages RcolorBrewer (Version 1.1-2), heatmap.plus (Version 1.3), and gplots (Version 3.1.1), as previously described [45]. For all heatmaps, Euclidean distance with ward.D2 clustering was used on the rows and columns. For IPA analysis, the color scale used represented the z-score that was exported directly from IPA. For all BSCE analyses, the color scales represented the −log(*p*-value). If there was a negative correlation, the −log(*p*-value) was multiplied by −1 to indicate suppression. Asterisks were placed if that cell was found to be statistically significant (−log(*p*-value) ≥ 4). For the biomarker heatmap, the −log(*p*-value) was shown on the cells that were significantly correlated. For FACTORIAL activation assays, the color represents the potency values (AC_50_) in µM that were reported in the original study [44].

## 3. Results

### 3.1. Structures of PFAS Chemicals Examined in the Study

**Figure 1:**
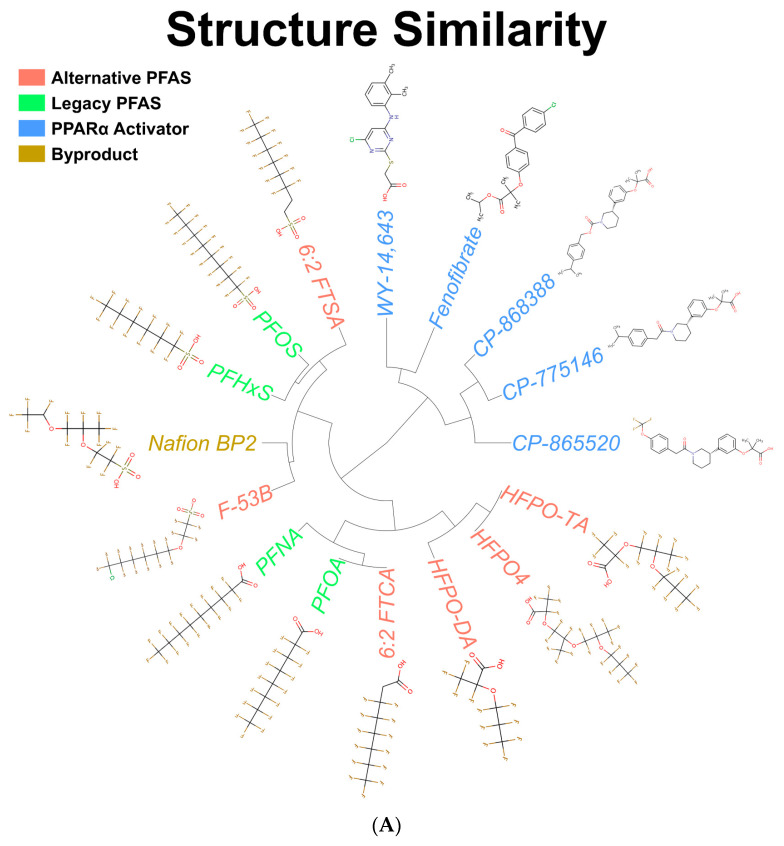

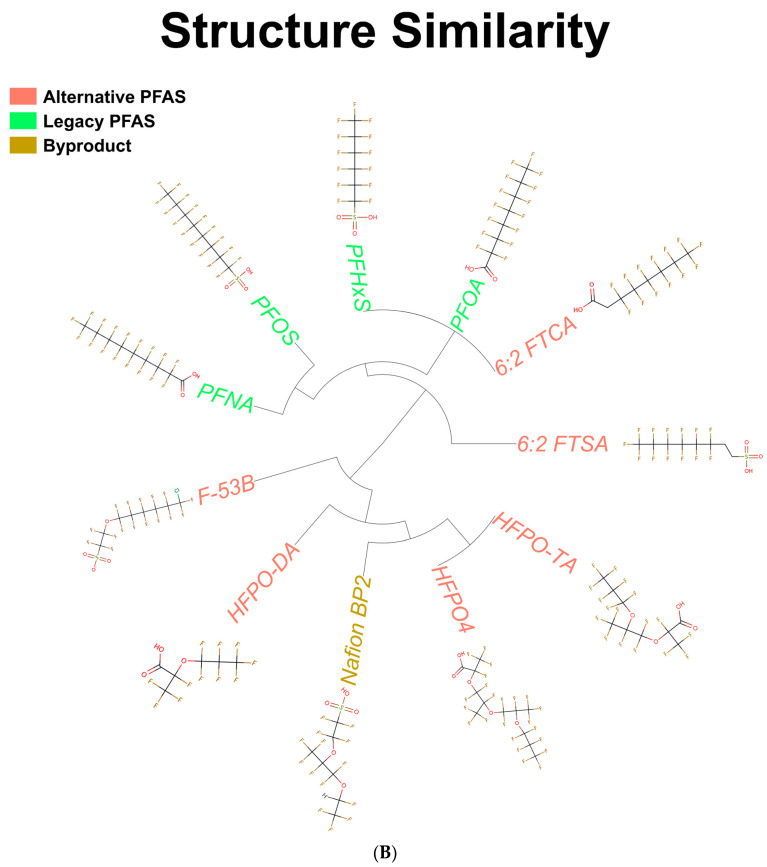

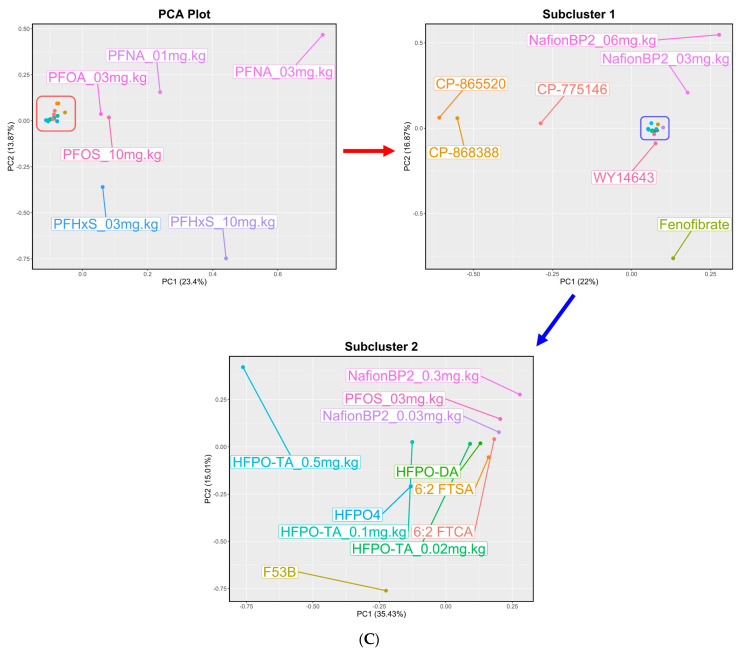
**Structural and transcriptional comparisons between the legacy and alternative PFAS.** (**A**) Unsupervised clustering of the ToxPrints for alternative PFAS, legacy PFAS, and PPARα activator compounds. (**B**) Unsupervised clustering of the ToxPrints for the PFAS compounds in the absence of their head group. (**C**) Principal component analyses of the transcriptomes for compounds examined in this study. The red box and blue box represent chemicals within subcluster 1 and subcluster 2, respectively. Each point represents a compound and dose, as labeled.

Unsupervised clustering analysis of the PFOA, PFOS, PFNA, PFHxS, 6:2 FTSA, 6:2 FTCA, Nafion BP2, F-53B, HFPO-DA, HFPO-TA, HFPO4, WY-14,643, fenofibrate, CP-868388, CP-775146, and CP-865520 ToxPrint features was performed to determine the structural similarities. The structures for each compound can be found in Table S1. The compounds with a sulfonic acid group (PFOS, PFHxS, 6:2 FTSA, Nafion BP2, and F-53B) or a carboxylic acid group (PFNA, PFOA, 6:2 FTCA, HFPO-DA, HFPO4, and HFPO-TA) clustered separately (Figure 1A). Given the similarities in their structures, all five PPARα activators clustered together, with WY-14,643 and fenofibrate being more similar to one another compared to the set of compounds designed to treat dyslipidemia (CP-868388, CP-775146, and CP-865520) [36]. To determine the similarities of each compound in the absence of the head group, a second clustering analysis was performed. The analysis separated the PFAS compounds into two main clusters, including one containing the four legacy PFAS, as well as 6:2 FTCA and 6:2 FTSA, and the other cluster containing the alternative PFAS (F-53B, HFPO-DA, HFPO4, and HFPO-TA) and Nafion BP2 (Figure 1B). The separation of the compounds appeared to be driven by the ether linkages in the fluorocarbon backbone; Nafion BP2 and alternative PFAS compounds have at least one ether linkage, whereas the legacy PFAS lack any ether linkages.

### 3.2. Identification of Genes Modulated by PFAS in the Mouse Liver

We compared transcript profiles derived from PFAS treatment examined under conditions that were as similar as possible to each other. All datasets examined were derived from the livers of male mice exposed daily through the oral route by gavage for 7 to 30 days (Table 1 and Table S1). The gene expression effects of reference PPARα activators were also examined in male mice treated by gavage and evaluated after either 6 h, 8 h, or 5 days. These are the only data available that approximate the exposure scenario of PFAS studies (Table S1).

Statistically filtered DEG lists were derived, as described in the Methods. A wide range in the number of DEGs was demonstrated across the treatment groups, with the PFNA treatment at 3 mg.kg^−1^ having the greatest number of significantly altered genes (9519 total) and 6:2 FTCA having the smallest number of DEGs (112 total) (Figure S2A). Principal Component Analysis (PCA) revealed that the legacy PFAS, PFNA at 1 and 3 mg.kg^−1^, PFOS at 10 mg.kg^−1^, PFOA at 3 mg.kg^−1^, and PFHxS at 3 and 10 mg.kg^−1^ exhibited the greatest differences compared to all the other PFAS and PPARα activators (Figure 1C, upper left). A sub-cluster analysis of the remaining compounds showed that the PPARα activators (CP-868388, CP-775146, CP-865520, WY-14,643, and fenofibrate) and Nafion BP2 at 3 and 6 mg.kg^−1^ were the next most dissimilar compounds (Figure 1C, upper right). A final subcluster was performed to visualize the similarities between the remaining PFAS; HFPO-TA at 0.5 mg.kg^−1^ and F-53B exhibited the least similar transcriptomic changes compared to the remaining 7 PFAS (Figure 1C, bottom).

### 3.3. Altered Canonical Pathways by PPARα Activators and PFAS

**Figure 2:**
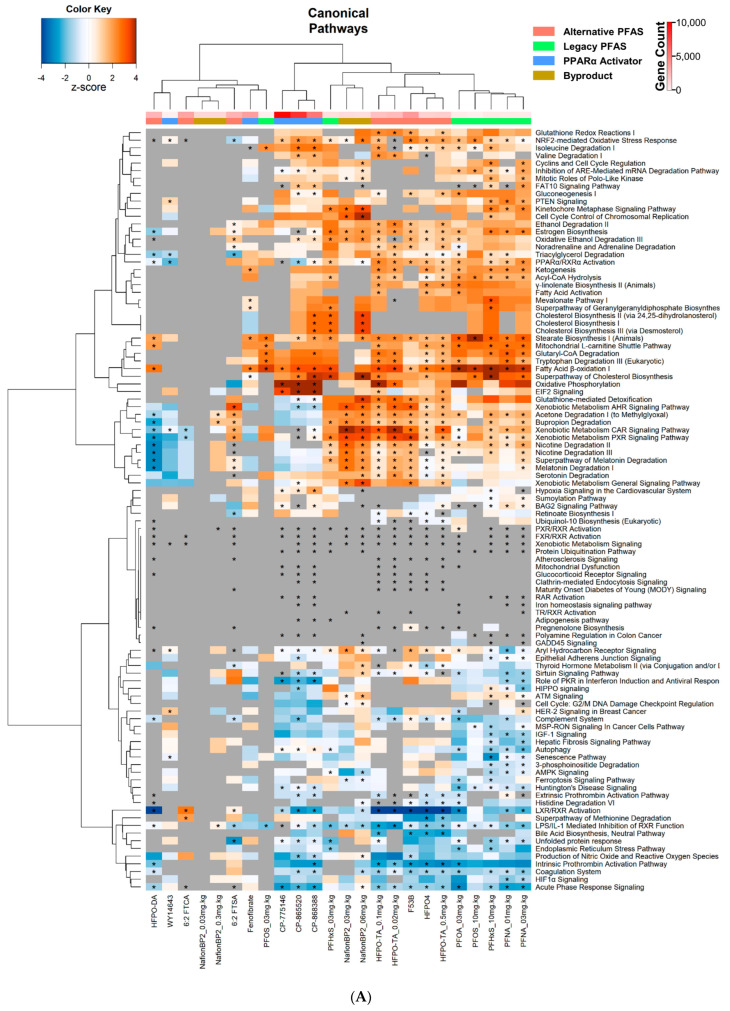

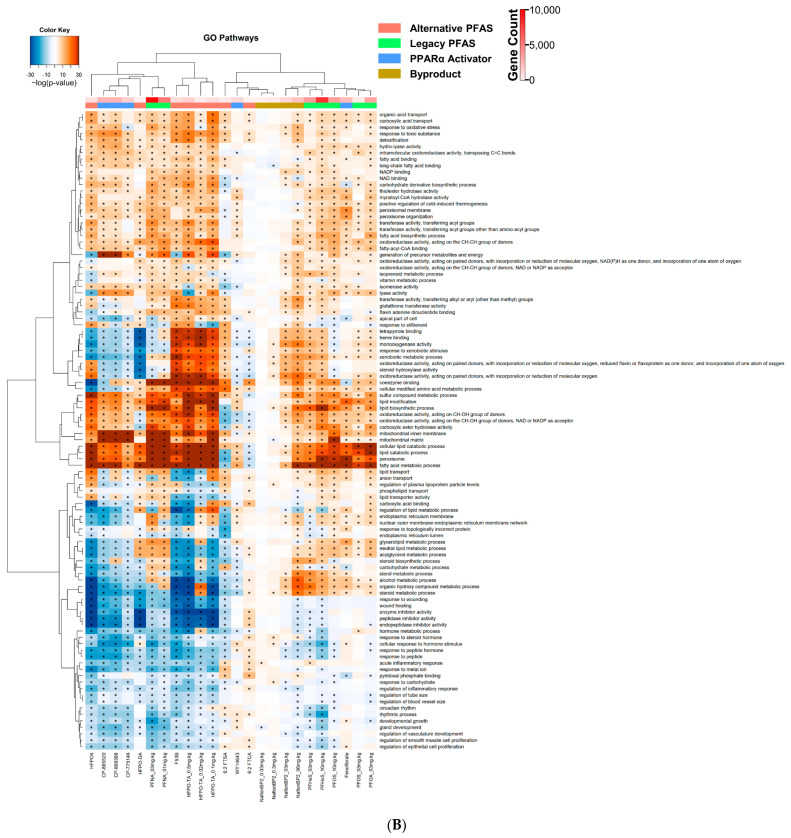

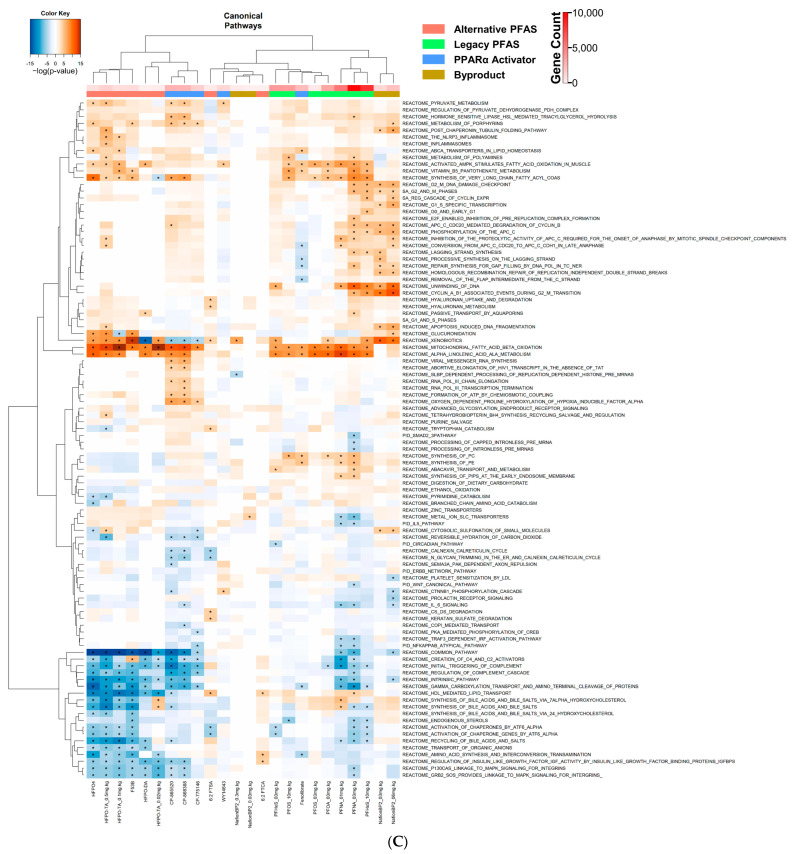
**Most alternative PFAS alter similar canonical pathways compared to legacy PFAS.** (**A**) Heatmap of the top 100 shared significantly altered canonical pathways exported from Ingenuity Pathway Analysis, with compounds and doses as columns and canonical pathways as rows. Heatmap color represents activation z-score, with a negative z-score indicating suppression and positive z-score indicating activation. An asterisk indicates a *p*-value ≤ 0.05. Grey cells that are significant indicate that IPA determined significant alterations in that pathway, however, could not assign a directional z-score. (**B**) Heat map of the top 100 altered, shared Gene Ontology (GO) pathways between legacy and alternative PFAS compounds. GO pathway analysis was carried out using BSCE. (**C**) Heat map illustrating the top 100 Broad Molecular Signatures Database (MSigDB) canonical pathways (from BSCE) that were significantly altered by PFAS treatment. Heatmap color represents −log(*p*-value) with values greater than and less than 0 indicating activation or suppression, respectively. An asterisk indicates if a −log(*p*-value) ≥ 4. If shown significant but also no color, this means that IPA could not identify direction but identified that the pathway was affected. For all heatmaps, red, green, and blue column color represents alternative PFAS, legacy PFAS, and PPARα activators, respectively. The red hue column color represents the number of genes within that treatment group.

Using Ingenuity Pathway Analysis (IPA), unsupervised clustering analysis was performed on the top 100 common altered canonical pathways across the PFAS treated groups (Table S2). Two large clusters existed: the first cluster exhibited little to no alterations, and the second cluster had many significant alterations in pathway activity (Figure 2A). The first cluster included HFPO-DA, 6:2 FTCA, 6:2 FTSA, and Nafion BP2 at 0.03 and 0.3 mg.kg^−1^. The second cluster was divided into three sub-clusters that separated compounds by chemical class. The first cluster contained the 3 PPARα activators, CP-868388, CP-775146, and CP-865520. The second subcluster contained the sulfonic acid PFAS PFHxS at 3 mg.kg^−1^ and Nafion BP2 at 3 and 6 mg.kg^−1^ (Figure 2A). The third subcluster contained the alternative PFAS, HFPO-TA at 0.02, 0.1, and 0.5 mg.kg^−1^, HFPO4, and F-53B, and the legacy PFAS, PFOA, PFOS at 3 mg.kg^−1^, PFNA at 1 and 3 mg.kg^−1^, and PFHxS at 10 mg.kg^−1^. The separation of the first subcluster from the others appears to be driven mainly by pathway differences between those activated by the PFAS and those suppressed by the PPARα activators. These pathways included ‘acetone degradation’, ‘bupropion degradation’, ‘nicotine degradation’, ‘melatonin degradation’, ‘glutathione-mediated detoxification’, ‘xenobiotic metabolism constitutive androstane receptor (CAR) and pregnane X receptor (PXR) signaling’, and ‘PPARα/RXRα activation’ (Figure 2A). The analysis indicates that many of the PFAS activate CAR and PXR, whereas the three CP PPARα activator compounds do not.

To further compare altered pathways, DEGs were analyzed by Gene Ontology (GO) enrichment in BaseSpace Correlation Engine (BSCE). Each gene list was compared to the library of GO gene lists using the Running Fisher test, as described in the Methods. The −log(*p*-value)s of the correlations were analyzed by unsupervised cluster analysis. The GO analysis is presented in Figure 2B and Table S3. The profiles were grouped into two main clusters. The first cluster contains CP-868388, CP-775146, CP-865520, HFPO-DA, HFPO4, F-53B, HFPO-TA at 0.02, 0.1, and 0.5 mg.kg^−1^, and PFNA at 1 and 3 mg.kg^−1^, with all other legacy PFAS, fenofibrate, Nafion BP2, 6:2 FTSA, 6:2 FTCA, and WY-14,643 found in the second cluster. Pathways that distinguish these two clusters included ‘glycerolipid metabolic process’, ‘neutral lipid metabolic process’, ‘acylglycerol metabolic process’, ‘steroid biosynthetic process’, ‘carbohydrate metabolic process’, ‘sterol metabolic process’, ‘alcohol metabolic process’, ‘organic hydroxy compound metabolic process’, and ‘steroid metabolic process’ which were mostly suppressed in cluster 1 and activated in cluster 2. Pathways, such as ‘monooxygenase activity’, ‘response to xenobiotic stimulus’, ‘oxidoreductase activity’, and ‘steroid hydroxylase activity’, were found to be significantly activated by Nafion BP2 at 3 and 6 mg.kg^−1^, and F-53B, HFPO-TA at 0.02, 0.1, and 0.5 mg.kg^−1^, and 6:2 FTSA but suppressed or not activated by the PPARα activators. Comparing legacy to alternative PFAS, ‘glycerolipid metabolic process’, ‘neutral lipid metabolic process’, ‘acylglycerol metabolic process’, ‘sterol metabolic process’, ‘alcohol metabolic process’, ‘organic hydroxy compound metabolic process’, and ‘steroid metabolic process’ tended to be activated in the legacy PFAS while suppressed in the alternative PFAS. The pathways ‘response to wound healing’, ‘wound healing’, ‘enzyme inhibitor activity’, ‘peptidase inhibitor activity’, and ‘endopeptidase inhibitor activity’ were either slightly suppressed or exhibited no changes in the legacy PFAS, while alternative PFAS had a stronger suppression.

We also examined the Broad Institute canonical pathways, consisting of the REACTOME and the Pathway Interaction Database (PID). The alternative PFAS compounds HFPO4, HFPO-TA at 0.02, 0.1, and 0.5 mg.kg^−1^, F-53B, and HFPO-DA clustered with the PPARα activators CP-865520, CP-868388, and CP-775146 (Figure 2C). 6:2 FTSA, 6:2 FTCA, WY-14,643, and Nafion BP2 at 0.03 and 0.3 mg.kg^−1^ had the least number of canonical pathways impacted (Figure 2C). The suppression of the ‘common pathway’, ‘Creation of C4 and C2 Activators’, ‘Initial triggering of Complement’, ‘Gamma Carboxylation Transport and Amino Terminal Cleavage of Proteins’, ‘Unwinding of DNA’, and ‘Cyclin A B1 associated Events During G2 M Transition’ were likely the main drivers of the separation of the clusters. In addition, HFPO4, HFPO-TA at 0.1 and 0.5 mg.kg^−1^, F-53B, PFHxS at 10 mg.kg^−1^ and PFNA at 3 mg.kg^−1^ all suppressed synthesis of bile acids and salts as exhibited in other studies [46].

### 3.4. Transcriptional Regulator and Factor Activation Status in PFAS and PPARα Activator Treated Mice

**Figure 3:**
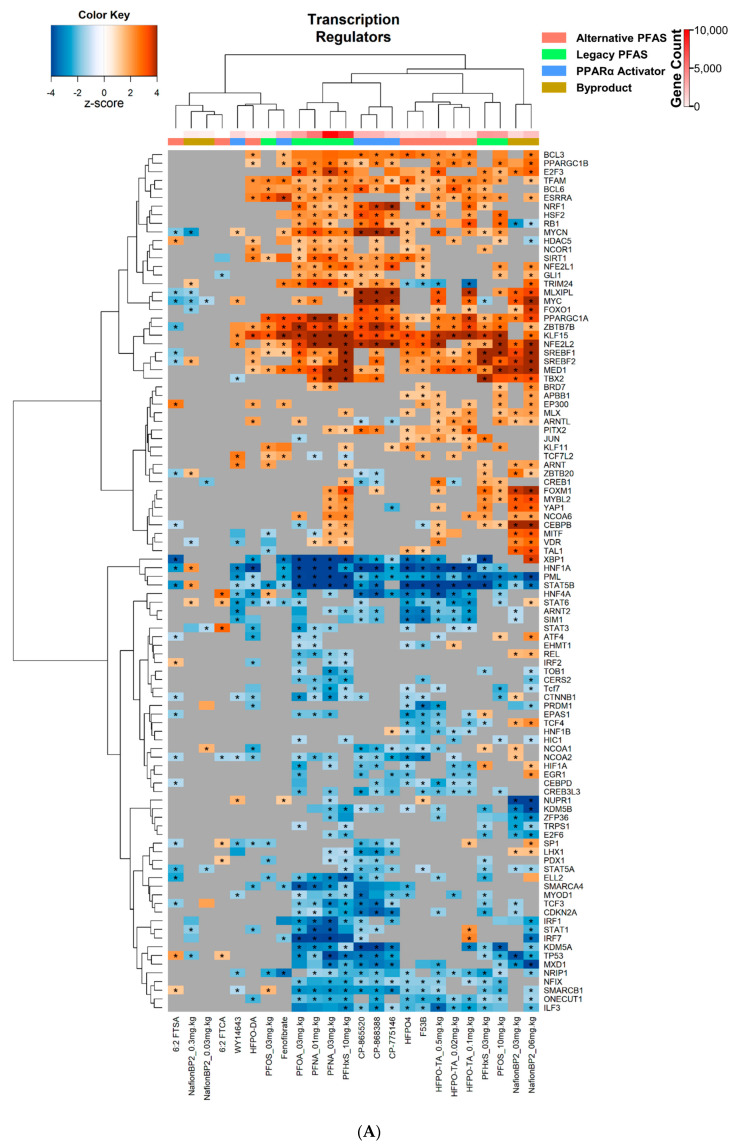

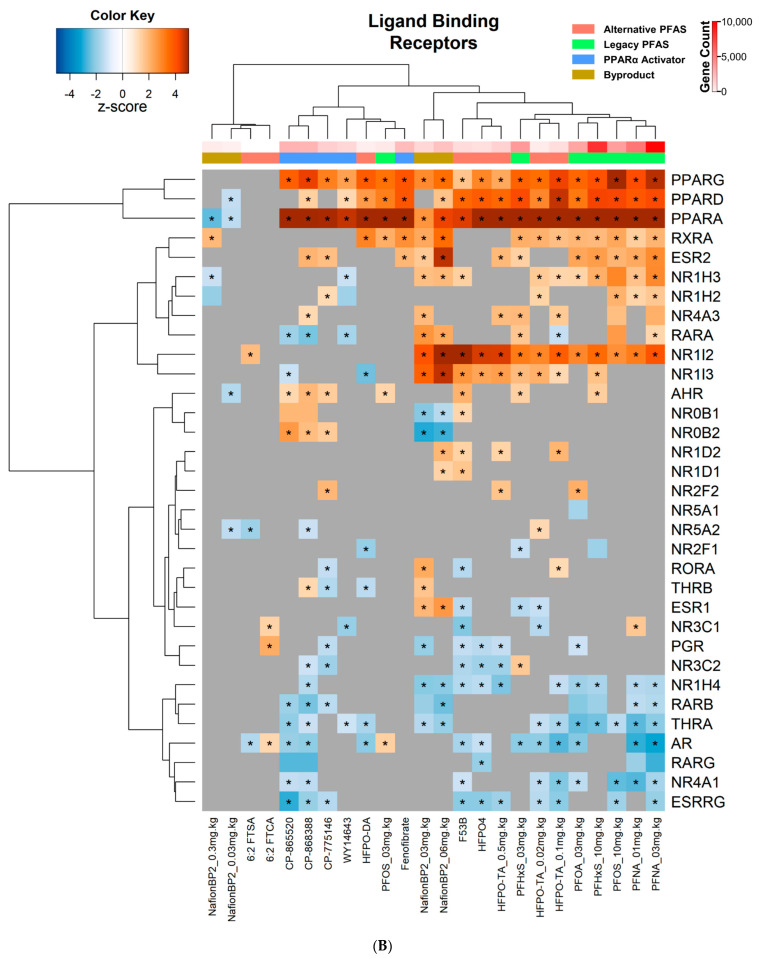
**Identification of transcriptional regulators predicted to be modulated by PFAS.** Heatmap of the activity of (**A**) non-nuclear receptor transcription factors and (**B**) ligand-binding nuclear receptors from IPA. The compounds in the columns and rows are the pathways. Red, green, and blue column colors represent alternative, legacy PFAS, and PPARα activators, respectively. The red hue column color represents the number of genes within that treatment group. Heatmap color represents activation z-score, with a negative z-score meaning suppression and positive z-score meaning activation. An asterisk indicates a *p*-value ≤ 0.05.

To determine alterations in the transcriptional regulators that drive the expression of the altered genes, an upstream analysis was carried out in IPA (Table S4). We found a positive exponential increase between the number of DEGs and the number of significantly altered transcriptional regulators, with one treatment being an outlier (NafionBP2 at 6 mg/kg) (Figure S2B). We first examined transcription factors that were not classified as “liganded binding receptors”. Cluster analysis was performed on the corresponding IPA activation Z-scores for each transcriptional regulator. Two main clusters formed, with one having few modulations, whereas the second having many significant modulations of transcriptional regulators (Figure 3A). The PFAS 6:2 FTSA, 6:2 FTCA, Nafion BP2 at 0.03 and 0.3 mg.kg^−1^, HFPO-DA, and PFOS at 3 mg.kg^−1^ had the fewest changes compared to the other PFAS, similar to the IPA canonical pathway analysis. Furthermore, the PPARα activators WY-14,643 and fenofibrate had fewer changes compared to CP-775146, CP-868388, and CP-865520. Within the second, more active and larger cluster, the alternative PFAS, HFPO4, HFPO-TA at 0.02, 0.1, and 0.5 mg.kg^−1^, and F-53B clustered together, while the legacy PFAS, PFNA at 1 and 3 mg.kg^−1^, PFHxS at 10 mg.kg^−1^, and PFOA at 3 mg.kg^−1^ clustered together. This clustering was partially driven by the alternative PFAS, which had significant suppression of the transcriptional regulators HNF4A, STAT6, ARNT2, SIM1, CEBPD, and CREB3L3, whereas the legacy PFAS exhibited significant suppression of MXD1, TP53, KDM5A, CDKN2A, and TCF3.

Using the upstream analysis function of IPA, transcriptional regulators classified as “liganded binding receptors” were analyzed. Other than AhR, all these receptors are members of the nuclear receptor superfamily. Unsupervised clustering analysis was performed on the activation Z-scores. There were two major clusters that separated the PFAS into those that activated the PPAR subtypes from those that had little, if any, PPAR subtype activation (Figure 3B). Those PFAS that did not activate PPAR subtypes included 6:2 FTSA, and 6:2 FTCA, as well as the two lower doses of Nafion BP2 (Figure 3B). Another major subcluster included PFAS that activated NR1I2 (PXR) (Nafion BP2 at 3 and 6 mg.kg^−1^, F-53B, HFPO4, HFPO-TA at 0.02, 0.1, and 0.5 mg.kg^−1^, PFNA at 1 and 3 mg.kg^−1^, PFOA at 3 mg.kg^−1^, PFOS at 10 mg.kg^−1^, and PFHxS at 3 and 10 mg.kg^−1^) and those that did not, which included the PPARα activators. The nuclear receptor NR1I3 (CAR) was activated by a subset of these treatments (Nafion BP2 at 3 and 6 mg.kg^−1^, HFPO4, F-53B, PFHxS at 3 and 10 mg.kg^−1^, and HFPO-TA at 0.02, 0.1, and 0.5 mg.kg^−1^). Additionally, the nuclear receptors AR and THRA were suppressed by HFPO-TA at 0.02 and 0.1 mg.kg^−1^, PFOA, PFNA at 1 and 3 mg.kg^−1^, HFPO-DA, CP-868388, and CP-865520. NR1H4 (FXR), a nuclear receptor important in bile acid metabolism in the liver, was found to be significantly suppressed in the legacy PFAS PFNA at 1 and 3 mg.kg^−1^, PFOA at 3 mg.kg^−1^, and PFHxS at 10 mg.kg^−1^ and in the alternative PFAS F53B, HFPO4, and HFPO-TA at 0.1 and 0.5 mg.kg^−1^. Likewise, the Nafion BP2 at 3 and 6 mg.kg^−1^ had significant suppression of FXR.

**Figure 4:**
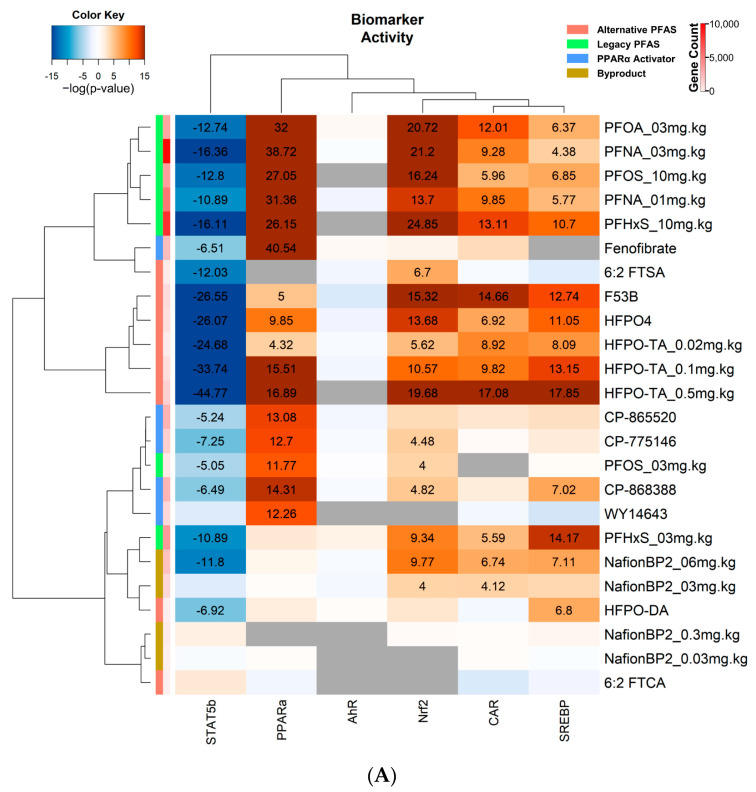

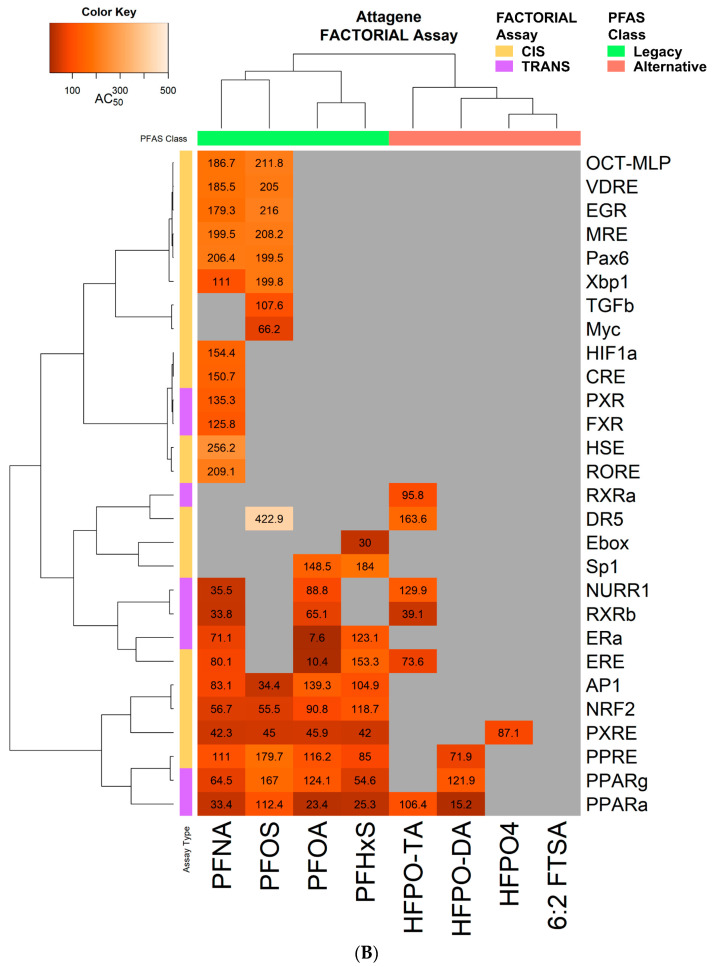
**Activity of transcription factors assessed using gene expression biomarkers or the Attagene Factorial assay.** (**A**) Each gene list was compared to the indicated biomarkers using the Running Fisher test in BSCE. The heatmap shows the resulting correlation −log(*p*-value)s. Shades of blue represent factor suppression, and shades of orange represent factor activation. Numbers show the −log(*p*-value)s for each correlation that was significant (|−Log(*p*-value)| ≥ 4). Column colors indicate the number of genes within that treatment group and the compound class. (**B**) Heatmap of potency values (AC_50_) for FACTORIAL activation assays. Color represents the AC_50_ in µM, number shows the exact AC_50_ reported in Houck et al. 2021, the row colors represent the PFAS class, and the column color represents the type of FACTORIAL assay.

The gene lists were compared to gene expression biomarkers predictive of the modulation of STAT5b, PPARα, AhR, NRF2, CAR, and SREBP (Table S5) [22,30,38,42,47,48]. As expected, all PPARα activators showed the activation of PPARα (Figure 4A). Most of the PFAS exhibited activation of PPARα, including PFNA at 1 and 3 mg.kg^−1^, PFOA at 3 mg.kg^−1^, PFHxS at 10 mg.kg^−1^, PFOS at 3 and 10 mg.kg^−1^, HFPO-TA at 0.02, 0.1, and 0.5 mg.kg^−1^, HFPO4, and F-53B. Nafion BP2 at all doses, HFPO-DA, 6:2 FTSA, and 6:2 FTCA exhibited no significant increases in PPARα activity. A separate 90-day subchronic HFPO-DA exposure study that was not part of our original analysis was examined using the PPARα biomarker [49]. In this study, the two highest doses (0.5 mg.kg^−1^ and 5 mg.kg^−1^) of HFPO-DA significantly activated PPARα, whereas 0.1 mg.kg^−1^ had no PPARα activity (Figure S3). SREBP activation occurred after exposure to PFNA at 1 and 3 mg.kg^−1^; PFHxS at 3 and 10 mg.kg^−1^; PFOS at 10 mg.kg^−1^; HFPO-TA at 0.02, 0.1, and 0.5 mg.kg^−1^; HFPO4, F-53B, Nafion BP2 at 6 mg.kg^−1^; and HFPO-DA (Figure 4A). PFNA at 1 mg.kg^−1^; PFHxS at 3 and 10 mg.kg^−1^; HFPO-TA at 0.02, 0.1, and 0.5 mg.kg^−1^; HFPO4, F-53B, PFOA at 3 mg.kg^−^; PFOS at 10 mg.kg^−1^; and Nafion BP2 at 3 and 6 mg.kg^−1^ exhibited CAR activation. Additionally, NRF2 was activated by all PFAS except HFPO-DA, 6:2 FTCA, and Nafion BP2 at 0.03 and 0.3 mg.kg^−1^ (Figure 4A). AhR activity was not altered by any PFAS or PPARα activator. Lastly, STAT5B was suppressed in all PFAS treatment groups except for Nafion BP2 at 0.03, 0.3, and 3 mg.kg^−1^, and 6:2 FTCA.

Lastly, we determined the overlap in targets in mouse liver predicted by computational techniques and those targets activated in human high throughput screens. We examined data derived from the Attagene FACTORIAL transcription screens [44] in which there are two types of assays carried out in HepG2 cells. The CIS assay utilizes a standard reporter driven by an endogenously expressed transcription factor. The TRANS assay utilizes an exogenously expressed hybrid protein consisting of the ligand binding domain of the nuclear receptor of interest fused in frame to the DNA binding domain of the yeast GAL4 transcriptional regulator [50]. These assays determine if tested compounds directly modulate receptor activity by physically interacting with the transcription factor ligand domain. Eight PFAS were analyzed as described above and in the Attagene assays: PFOS, PFOA, PFNA, PFHxS, HFPO-DA, HFPO-TA, HFPO4, and 6:2 FTSA. We performed a clustering analysis of the potency values (AC_50_) using the data set generated from Houck et al. 2021 (Figure 4B) [44]. Clustering separated the four legacies from the four alternative PFAS. The separation of the clusters was likely driven by the activation of a number of transcription factors or activation of response elements by the legacy PFAS but not the alternative PFAS. It should be noted that 6:2 FTSA did not seem to activate any nuclear receptors in this screen. PPARα was activated by PFNA, PFOS, PFOA, PFHxS, and HFPO-DA, which were also detected by the predictive mouse biomarkers (Figure 4A,B). Interestingly, HFPO4 did not activate PPARα in either the CIS or TRANS activation assays but activated PPARα in mouse liver as determined by the biomarker and IPA analysis. For NRF2 activation, only the legacy PFAS PFNA, PFOS, PFOA, and PFHxS all activate NRF2 directly, suggesting that the activation of HFPO-TA, HFPO4, and 6: FTSA is through indirect mechanisms (Figure 4A,B).

## 4. Discussion

The ~12,000 PFAS compounds in commerce include newer alternative PFAS with structural changes from the legacy PFAS that could potentially lead to increased metabolism and excretion, causing a decrease in bioaccumulation. This leads to the hypothesis that alternative PFAS may be less toxic. As one way to test this hypothesis, we compiled and analyzed male mouse hepatic gene expression profiles after exposure to 11 PFAS from multiple in vivo studies (Table 1, Figure S1) to identify similarities and differences between legacy and alternative PFAS in the molecular targets important in toxicity, especially in the liver. Application of pathway and transcription factor analysis methods, including the use of well characterized predictive gene expression biomarkers, led to a wealth of findings for these PFAS. Remarkably, there appears to be no major difference in the most prominent transcriptional targets between the legacy PFAS, which have well characterized toxicities, and the alternative PFAS, in which knowledge of toxicity is more limited. Both legacy and alternative PFAS activated PPARα, CAR, Nrf2, and SREBP, while suppressing STAT5b and having no effect on AhR. Only the alternative PFAS 6:2 FTCA and 6:2 FTSA appeared to have a minimal impact on the hepatic transcriptome. Modulation of the activity of these transcription factors indicates that exposure to PFAS may lead to activation of pathways governing endocrine disruption, liver steatosis, and cancer. The results imply that, in general, the alternative PFAS examined here activate similar toxicity pathways as the legacy PFAS.

### 4.1. Effects of PFAS on Bile Acid Metabolism

Bile acid content is a commonly used biomarker for liver injury. Although the bile acid metabolism is a process of hepatoenteric circulation, FXR in the mouse liver is a key NR for bile acid metabolism, which influences synthesis and reabsorption of bile acid via regulating CYP27A1 and 7A1. In earlier studies, PFAS increased total bile acids and inhibited FXR [51]. Our analysis utilizing IPA found that the legacy PFAS PFNA at 1 and 3 mg.kg^−1^, PFOA at 3 mg.kg^−1^, and PFHxS at 10 mg.kg^−1^ and the alternative PFAS F53B, HFPO4, and HFPO-TA at 0.1 and 0.5 mg.kg^−1^ suppressed FXR. Additionally, Nafion BP2 at 3 and 6 mg.kg^−1^ had significant suppression of FXR. Therefore, including lipid metabolism, changes in pathways that relate to bile acid metabolism are also worth investigating in further studies.

### 4.2. Effects of PFAS on PPARα Activity

PPAR family members are arguably the best characterized targets of legacy PFAS; less is known about the effect of alternative PFAS on PPAR activity. The analysis of the transcript profiles using the IPA upstream analysis indicated that all treatments except the two low doses of Nafion BP2 and 6:2 FTCA and 6:2 FTSA activated PPARα and PPARγ and in most cases, PPARβ. While IPA analysis can give us clues about the transcription factors that regulate the differentially expressed genes, we have previously shown that the genes annotated as PPARα-regulated in IPA are not necessarily PPARα-dependent by showing that these same genes are similarly expressed in the livers of wild-type and PPARα-null mice after exposure to PPARα activators (Corton et al., submitted). Given that the biomarker approach has excellent accuracy in identifying PPARα activators [42], we ultimately based our conclusions of PPARα activity on the biomarker approach. Consistent with past studies [42], we show that the legacy PFAS PFOS, PFOA, PFHxS, and PFNA, as well as the four known PPARα activators examined, activated PPARα. The alternative PFAS HFPO-TA at all three doses tested, F53B, and HFPO4 were found to activate PPARα. At least for HFPO-TA and HFPO4, past studies confirmed PPARα activity by RT-qPCR, showing increased expression of PPARα marker genes (e.g., *Cyp4a10*, *Cyp4a14*, *Cd36*) [34,35]. The conclusions are supported by results of the human Attagene assays in which PPARα was activated by PFNA, PFOS, PFOA, PFHxS, and HFPO-DA.

It was initially surprising that HFPO-DA at 1 mg.kg^−1^ for 28 days did not significantly activate PPARα when analyzed using the PPARα biomarker. We analyzed a subchronic 90-day HFPO-DA exposure that included three doses [49] and found that HFPO-DA induces PPARα at the daily doses of 0.5 and 5 mg.kg^−1^ but not 0.1. This suggests that 1 mg.kg^−1^ daily exposure for 28 days may not be sufficient to induce PPARα compared to a 90-day exposure. These results are consistent with the fact that HFPO-DA is a relatively weak PPARα activator in rats with doses up to 250–500 mg.kg^−1^ needed to achieve maximum activation of PPARα-dependent genes in the fetal or maternal liver [52,53].

Three PFAS were examined with no clear evidence of PPARα activation: 6:2 FTCA, 6:2 FTSA, and Nafion BP2. Nafion BP2 was examined at doses up to 6 mg.kg^−1^ with no activation, as shown using the PPARα biomarker despite robust activation of other transcription factors (discussed below). There was some indication that Nafion BP2 could activate PPARα at 3 and 6 mg.kg^−1^, as there was increased expression of genes that were thought to be regulated by PPARα. An extensive analysis comparing the genes altered by legacy PFAS in wild-type and PPARα-null mice found that the genes regulated by Nafion BP2 were regulated by the legacy PFAS in both wild-type and PPARα-null mice. Because these genes are regulated in the absence of an intact PPARα, this analysis indicates that Nafion BP2 activates other PPAR subtypes, which can regulate these genes through a similar mechanism as all PPAR subtypes regulate gene expression through the direct repeat 1 DNA response element. The absence of PPARα activation by Nafion BP2 is consistent with little to no activation in rat maternal dams at doses up to 30 mg.kg^−1^ [16].

Also, 6:2 FTCA and 6:2 FTSA did not activate PPARα as assessed using the PPARα biomarker. These results were somewhat surprising, considering that these compounds are structurally very similar to PFOA and PFOS, except that they lack any fluorination at the first (6:2 FTCA) or first and second (6:2 FTSA) carbon adjacent to the head group. The dose used in this study (5 mg.kg^−1^) was comparable to doses of PFOA and PFOS that activated PPARα in previous studies. In the original study describing the effects of these PFAS [15], the authors found minimal increases in the expression of PPARα target genes, while there were increases in inflammatory mediators in the serum by 6:2 FTSA, including tumor necrosis factor alpha, interleukin 1 beta, and interleukin 10. Because only one dose of the chemicals was tested, it is possible that PPARα would be activated at higher doses.

Adverse phenotypic outcomes associated with hepatic PPARα activation include hepatomegaly, alteration in lipid levels, increases in hepatocyte proliferation, and increases in liver tumors in rodents [19]. While no 2-year chronic assays have been conducted for any PFAS in mice, chronic activation of PPARα in rats by PFOA, PFOS, and GenX (HFPO-DA) induced tumors in one or more targets in the “tumor triad” consisting of liver, testis, and pancreas (Corton et al. submitted). Extensive analysis by multiple workgroups has concluded that, at least for liver tumors, the PPARα mode of action is not or is likely not relevant to humans [19].

### 4.3. Effects of PFAS on CAR, PXR, and Nrf2 Activity

Another common target of legacy PFAS in rodent livers is the nuclear receptor CAR. Many studies have shown that PFOA, PFOS, PFNA, and PFHxS activate CAR using either trans-activation assays or are implied by observing the activation of CAR-regulated genes [22,25,33]. Our previous studies using a CAR biomarker showed that not only did the legacy PFAS activate CAR, but the activation was greater in the absence of PPARα in *Ppara*-null mice compared to wild-type mice [22]. In the present study, we not only confirmed that the legacy PFAS activated CAR, but that the alternative PFAS, including F53B, HFPO4, and all dose levels for HFPO-TA, were activators of CAR. In addition, Nafion BP2 activated CAR at the two highest doses. Only 6:2 FTSA and 6:2 FTCA did not activate CAR. None of the PPARα activators activated CAR, but all of these chemicals had shorter exposure windows compared to the PFAS exposures. There is evidence that some PPARα activators that are not PFAS activate CAR after prolonged exposures [22]. Our results with the biomarker were generally consistent with the results of the IPA upstream analysis. However, the IPA analysis did not find that all legacy PFAS activated CAR. When investigating the FACTORIAL human trans-activation, none of the PFAS in our analysis showed CAR activation, indicating species differences in response.

Activation of CAR leads to alteration in the expression of phase 1 and phase 2 drug metabolizing enzymes, hepatocyte hypertrophy, and proliferation, and after chronic exposure, hepatocellular adenomas and carcinomas [54]. The role of CAR in mediating any carcinogenic effects of PFAS is unknown. After a 7-day exposure to PFOA or PFOS in wild-type and CAR-null mice, there were similar increases in liver to body weights and Ki67 labeling of hepatocytes, a measure of cell proliferation [22]. Taken together, PPARα likely plays a dominant role in mediating the activation of key events associated with liver tumor induction observed for PFOA. The relative contribution of CAR and PPARα to liver carcinogenesis for the other PFAS is unknown.

The IPA upstream analysis found that most of the PFAS led to the activation of PXR. While there is no biomarker that accurately predicts PXR activation, there is evidence from other studies that PXR is activated under some exposure scenarios. PFOA, PFOS, and PFHxS have been shown to exhibit PXR activity in rodents and lack activity in humans [23,55,56]. Other PFAS, such as PFNA, are known as PXR activators in both humans and rodents [24,57]. Further evidence using FACTORIAL analysis shows that PFNA, PFOA, PFOS, PFHxS, and HFPO4 directly activate PXR, with PFHxS having the lowest AC_50_ [44].

In parallel with the activation of CAR and PPARα, we found that oxidant-induced Nrf2 was activated. Nrf2 was activated by all chemicals that activated either PPARα or CAR except those treatments that were only 6 h, which included the PPARα activators. Increases in oxidative stress and associated indirect DNA damage are thought to play a role in the PPARα mediated liver cancer mode of action (MOA) [18]. We previously found a strong relationship between the activation of PPARα, CAR, and AhR upstream of Nrf2 activation [24]. The only treatment in which there was Nrf2 activation in the absence of either PPARα or CAR activation was after exposure to 6:2 FTSA. In this case, the increases in Nrf2 could be due to increases in oxidative stress in parallel with the increases in liver damage and inflammation that were not seen after exposure to 6:2 FTCA, in which liver damage and inflammation were muted compared to 6:2 FTSA [15].

### 4.4. Effects of PFAS on SREBP1/2 Activity

We found that SREBP was activated in many of the exposure conditions examined. Sterol regulatory element binding proteins (SREBPs) are endoplasmic reticulum (ER) bound transcription factors that, when activated, regulate the expression of enzymes involved in fatty acid and cholesterol synthesis [58]. The SREBP family consists of three factors (SREBP1a, SREBP1c, and SREBP2) encoded on two genes. Each factor plays a unique role in lipid synthesis: SREBP2 regulates cholesterol synthesis, SREBP1c regulates fatty acid synthesis, and SREBP1a regulates both cholesterol and fatty acid synthesis [58]. Dysregulation of these transcription factors can drive lipid accumulation in the liver, leading to steatosis [59]. A previously described biomarker accurately detects the activity of SREBP family members [30]. We used this biomarker in the present study to understand the relationships between SREBP activation and steatosis, which is often seen in the livers of mice after exposure to PFAS.

Using the biomarker, we confirmed that the legacy PFAS PFHxS at 3 and 10 mg.kg^−1^, PFNA at 1 and 3 mg.kg^−1^, and PFOS only at 10 mg.kg^−1^ activated SREBP. This was supported by the IPA upstream analysis, in which PFOS at 10 mg.kg^−1^, PFHxS at 3 and 10 mg.kg^−1^, and PFNA at 1 and 3 mg.kg^−1^ activated SREBP1 and SREBP2. Exposure to legacy PFAS in the livers of treated mice leads to increases in triglyceride levels, a measure of steatosis [60]. There is evidence that PFOA can directly regulate the activity of SREBP by promoting the formation of active forms of SREBP [29]. However, it could be possible that SREBP activation occurs indirectly through PPARα by changes in the lipid composition of the ER and the release of active SREBPs through increasing the unsaturated to saturated fatty acid ratio [61]. Nonetheless, more studies are needed to determine how PFAS causes SREBP activation.

In addition to the legacy PFAS activating SREBP, we found that F53B, HFPO4, HFPO-DA, HFPO-TA at all three doses, and Nafion BP2 at the highest dose activated SREBP. For Nafion BP2, we found that SREBP was activated in parallel with increases in liver triglycerides (Corton et al., submitted). Both HFPO4 and HFPO-DA were found to cause mild increases in steatosis and necrosis [34]. Interestingly, the increases in SREBP as assessed using the biomarker, as well as the level of liver damage, were greater in HFPO4 than in HFPO-DA exposed mice [34]. Apparently, measures of steatosis have not been measured in the livers of mice exposed to F53B or HFPO-TA. However, F53B is thought to have the capability of inducing steatosis through PPARγ [62]. Our analysis indicates that activation of SREBP is a common target of PFAS and activation is found in the livers of all 7 legacy and alternative PFAS that cause steatosis, but not after exposure to perfluorobutane sulfonate (PFBS) [60] that does not cause steatosis. Further work is needed to determine whether SREBP activation is mechanistically linked to increases in triglyceride levels in the livers of treated mice.

### 4.5. Effects of PFAS on STAT5b Activity

Males and females exhibit differences in molecular phenotypes, such as the levels of lipid and drug metabolism enzymes [63]. The major pathway that regulates sexually dimorphic gene expression in the liver is the growth hormone (GH)-STAT5b signaling axis [64]. This axis is highly regulated by circulating GH levels. Here, we found that all the PFAS except 6:2 FTCA suppressed STAT5b. The STAT5b biomarker used in the analysis was built from male vs. female comparisons from the mouse liver and was ultimately filtered for those genes that were dependent on STAT5b for regulation [47]. Using the biomarker, we previously found that female mice treated with testosterone will cause increases in STAT5b, while treatment of male mice with estrogen will cause suppression of STAT5b. Thus, the suppression of STAT5b that we observed in the male mice was similar to that of feminization of the liver transcriptome. Feminization can occur through a number of mechanisms by disrupting the hypothalamic-pituitary-liver axis [47]. The fact that almost all of the PFAS examined in this study suppressed STAT5b indicates that there may be a common mechanism that underlies feminization. Further work is needed to determine if STAT5b is suppressed because of changes in the testosterone-estrogen balance or if the mechanism may be due to disruptions in growth hormone pulsatile release from the pituitary gland. Because the sexually dimorphic GH secretion pattern has a major impact on the expression of xenobiotic genes [47], we hypothesize that the livers of male mice treated with PFAS exhibit a pattern of xenobiotic metabolism similar to that of females.

### 4.6. Study Limitations

Our in silico approach, while efficient for identifying similarities of pathways, has its limitations. The approach offers preliminary insights but might not capture the full spectrum of biological complexities. Therefore, these results should be interpreted as initial indicators, underscoring the need for further experimental validation. A balanced integration of computational predictions followed by hypothesis testing is the best method for robust conclusions.

## 5. Conclusions

This comparative analysis of a variety of different legacy and alternative PFAS, along with a PFAS byproduct, shows similarities in the mechanisms of nuclear receptor action in the livers of mice. We confirmed the findings from past studies and identified a number of molecular targets that have not been previously discussed (Table 2). We showed that PPARα is activated by PFOS, PFOA, PFNA, PFHxS, HFPO-DA, HFPO4, HFPO-TA, and F-53B, similarly found in other studies. Moreover, we corroborated other studies showing that PFNA, PFHxS, PFOS, and PFOA activate NRF2 and found that Nafion BP2, HFPO-DA, HFPO4, HFPO-TA, and 6:2 FTSA are predicted to be NRF2 activators. We found that HFPO4, HFPO-TA, and F-53B are all CAR activators, and our results support other studies that found PFOS, PFOA, PFNA, PFHxS, and Nafion BP2 to be CAR activators. The results of the study reinforce the evidence that SREBP1/2 is activated and STAT5b is suppressed by PFOS, PFOA, PFNA, and PFHxS. Lastly, we found that Nafion BP2, HFPO-DA, HFPO4, HFPO-TA, and F-53B had similar effects across PPARα, NRF2, CAR, SREBP1/2, and STAT5b activity. Taken together, our work highlights the similarities in molecular targets between the legacy and alternative PFAS.

## Figures and Tables

**Table 1:** Compiled Studies Examined.

Chemical Name	Abbreviation	DTXSID	PMID	Dose	Timepoint
Perfluorooctane sulfonate	PFOS	DTXSID3031864	20936131	Daily 3 mg/kg or 10 mg/kg	7 days
Perfluorooctanoic acid	PFOA	DTXSID8031865	18281256	Daily 3 mg/kg	7 days
Perfluorononanoic acid	PFNA	DTXSID8031863	28558994	Daily 1 mg/kg or 3 mg/kg	7 days
perfluorohexane sulfonate	PFHxS	DTXSID7040150	28558994	Daily 3 mg/kg or 10 mg/kg	7 days
Perfluoro-2-([perfluoro-3-(perfluoroethoxy)-2-propanyl]oxy)ethanesulfonic acid	Nafion BP2	DTXSID10892352	#	Daily 0.03, 0.3, 3, or 6 mg/kg	7 days
Ammonium perfluoro-2-methyl-3-oxahexanoate	HFPO-DA (HFPO2) (GenX)	DTXSID40108559	27553808 32138627	Daily 1 mg/kg Daily 0.1, 0.5, or 5 mg/kg	28 days 90 days
Perfluoro-(2,5,8-trimethyl-3,6,9-trioxadodecanoic)acid	HFPO4	DTXSID70276659	27553808	Daily 1 mg/kg	28 days
Perfluoro-2,5-dimethyl-3,6-dioxanonanoic acid	HFPO-TA	DTXSID00892442	29927593	Daily 0.02, 0.1, 0.5 mg/kg	28 days
Potassium 9-chlorohexadecafluoro-3-oxanonane-1-sulfonate	F-53B	DTXSID60881236	#	Daily 5 mg/kg	28 days
6:2 Fluorotelomer sulfonic acid	6:2 FTSA	DTXSID6067331	28032147	Daily 5 mg/kg	28 days
2-Perfluorohexyl ethanoic acid	6:2 FTCA	DTXSID50472556	28032147	Daily 5 mg/kg	28 days
(S)-2-methyl-2-(3-(1-(2-(4-(trifluoromethoxy)phenyl)acetyl)piperidin-3-yl)phenoxy)propanoic acid sodium salt	CP-865520	DTXSID4044032	18971326	Daily 1 mg/kg	5 days
(S)-2-(3-(1-(2-(4-isopropylphenyl)acetyl)piperidin-3-yl)phenoxy)-2-methylpropanoic acid sodium salt	CP-775146	DTXSID9044033	18971326	Daily 1 mg/kg	5 days
(S)-2-(3-(1-((4-isopropylbenzyloxy)carbonyl)piperidin-3-yl)phenoxy)-2-methylpropanoic acid sodium salt	CP-868388	DTXSID4044034	18971326	Daily 1 mg/kg	5 days
Propan-2-yl 2-[4-(4-chlorobenzoyl)phenoxy]-2-methylpropanoate	Fenofibrate	DTXSID2029874	18301758	Single 4 mg/mL	6 h
([4-Chloro-6-(2,3-dimethylanilino)pyrimidin-2-yl]sulfanyl)acetic acid	WY-14,643	DTXSID4020290	26215100	Single 250 mg/kg	8 h

# Not Published.

**Table 2:** Summary of transcription factor modulation by the PFAS examined.

	Transcription Factor Activation or Suppression				
	PPARα	NRF2	CAR	SREBP1/2	STAT5b
PFOS	+ *	+ *	+ *	+ *	- *
PFOA	+ *	+	+ *	+ *	- *
PFNA	+ *	+	+ *	+ *	- *
PFHxS	+ *	+	+ *	+ *	- *
Nafion BP2	^NA^	+	+ *	+	-
HFPO-DA	+ *	+	^NA^	+	-
HFPO4	+ *	+	+	+	-
HFPO-TA	+ *	+	+	+	-
F-53B	+ *	^NA^	+	+	-
6:2 FTSA	^NA^	+	^NA^	^NA^	-
6:2 FTCA	^NA^	^NA^	^NA^	^NA^	^NA^

+ = activation; * = shown in previous studies; - = suppression; ^NA^ = no activity changed.

## Data Availability

Data are contained within the article and supplementary materials.

## Supplementary Materials

The following supporting information can be downloaded at https://www.mdpi.com/article/10.3390/toxics11120963/s1, Figure S1: Scheme of experimental workflow. Scheme providing a detailed representation of the strategy used in this study. Starting with a curated list of 11 PFAS and 5 PPARα actives, the subsequent steps involved the analysis of available transcript profile data. This data was thoroughly processed and then utilized for PCA, IPA, and BSCE. Other analyses include results for ToxPrints and Attagene FACTORIAL Assays that were systematically clustered to underline the similarities and differences observed among the compounds; Figure S2: Relationship of number of altered genes, pathways, and their respective *p*-values from all datasets. (A) Bar plot of the total number of significant DEGs (±1.2 fold change; *p*-value < 0.05) for each treatment group. Red and blue bars indicate the number of up- and down-regulated DEGs, respectively. The number above each bar is the total number of DEGs per group. The color of the treatment group labels represents the class of the compound. (B) Relationships between the number of genes and transcription factors altered. Scatterplot containing each dataset where the y axis corresponds to the number of significantly altered genes (±1.2 fold change; *p* < 0.05), and the x-axis corresponds to the number of significantly altered pathways (utilizing a ±2 z-score cutoff). The size of the bubble represents the number of significantly altered pathways (*p*-value < 0.05) and the color represents each compound; Figure S3: HFPO-DA induces PPARα activity in mice after a 90-day exposure. The dataset GSE135943 was analyzed through BSCE. In this study, three dose levels of HFPO-DA were administered to male mice by daily gavage for 90 days. RNA-Sequencing was performed on formalin fixed paraffin embedded liver tissues as described in the original study [49]. Each DEG list was compared to the PPARα biomarker in BSCE using the Running Fisher test. The *p*-values were exported and −log transformed. A bar plot is shown of the −log(*p*-value)s with the doses of HFPO-DA on the x-axis. The number above the bar is the −log(*p*-value); Table S1: Compound Information; Table S2: IPA Canonical Pathways Data; Table S3: BSCE Data; Table S4: IPA Upstream Regulators Data; Table S5: Predictive Biomarker Data.
