# Supplementary material for: Hepatic Transcriptome Comparative In Silico Analysis Reveals Similar Pathways and Targets Altered by Legacy and Alternative Per- and Polyfluoroalkyl Substances in Mice
Source: Toxics. 2023 Nov 28;11(12):963. doi: 10.3390/toxics11120963 (PMC10748317; doi:10.3390/toxics11120963)
Supplement: Supplementary file 1 [file toxics-11-00963-s001.zip › Supplemental Figures.pdf]

Supplemental Figures.

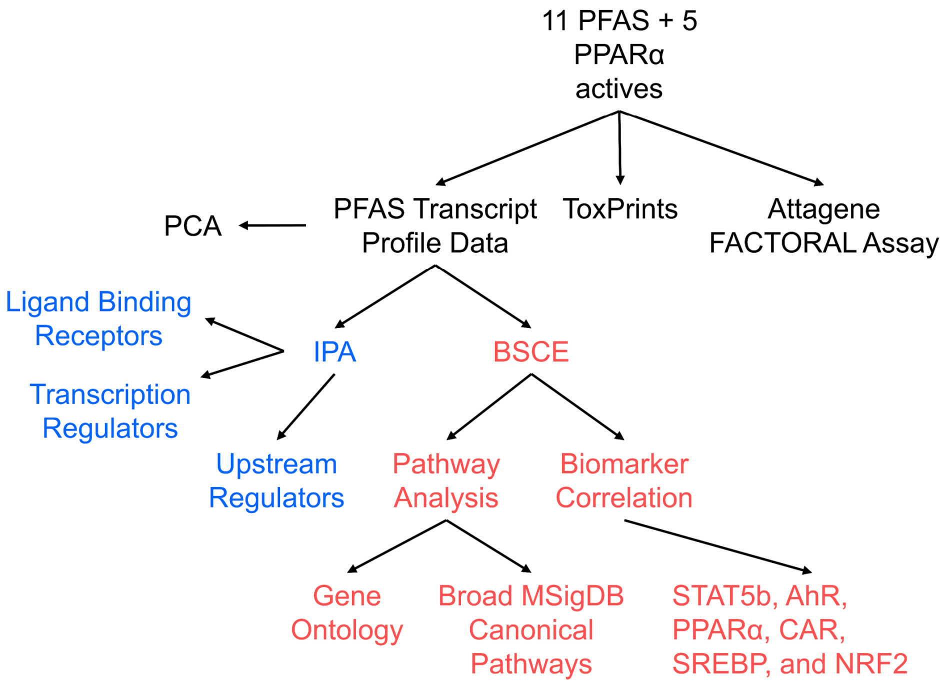

Figure S1: Scheme of experimental workflow. Scheme providing a detailed representation of the strategy used in this study. Starting with a curated list of 11 PFAS and 5 PPARα actives, the subsequent steps involved the analysis of available transcript profile data. This data was thoroughly processed and then utilized for PCA, IPA, and BSCE. Other analyses include results for ToxPrints and Attagene FACTORIAL Assays that were systematically clustered to underline the similarities and differences observed among the compounds.

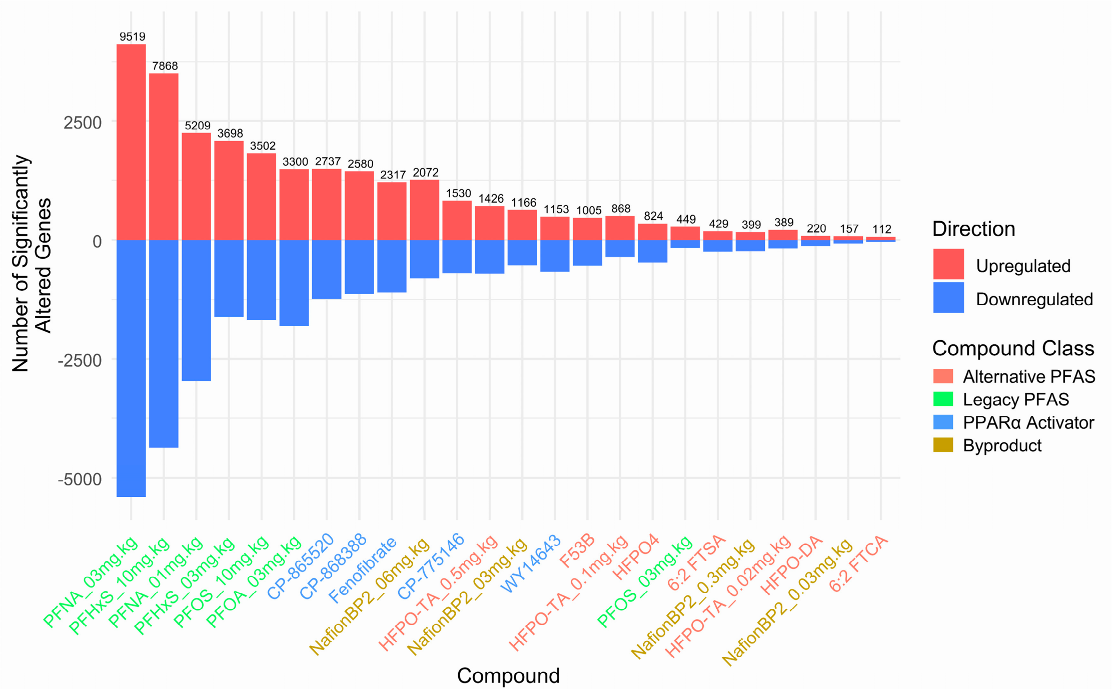
Number of Significantly Altered Genes
2500
0
-2500
-5000
9519
7868
5209
3698
3502
3300
2737
2580
2317
2072
1530
1426
1166
1153
1005
868
824
449
429
399
389
220
157
112
PFNA_03mg.kg
PFHxS_10mg.kg
PFNA_01mg.kg
PFHxS_03mg.kg
PFOS_10mg.kg
PFOA_03mg.kg
CP-865520
CP-868388
Fenofibrate
NafionBP2_06mg.kg
CP-775146
HFPO-TA_0.5mg.kg
NafionBP2_03mg.kg
WY14643
F53B
HFPO-TA_0.1mg.kg
HFPO4
PFOS_03mg.kg
6:2 FTSA
NafionBP2_0.3mg.kg
HFPO-TA_0.02mg.kg
HFPO-DA
NafionBP2_0.03mg.kg
6:2 FTCA
Compound
Direction
Upregulated
Downregulated
Compound Class
Alternative PFAS
Legacy PFAS
PPARα Activator
Byproduct

Fig S2A.

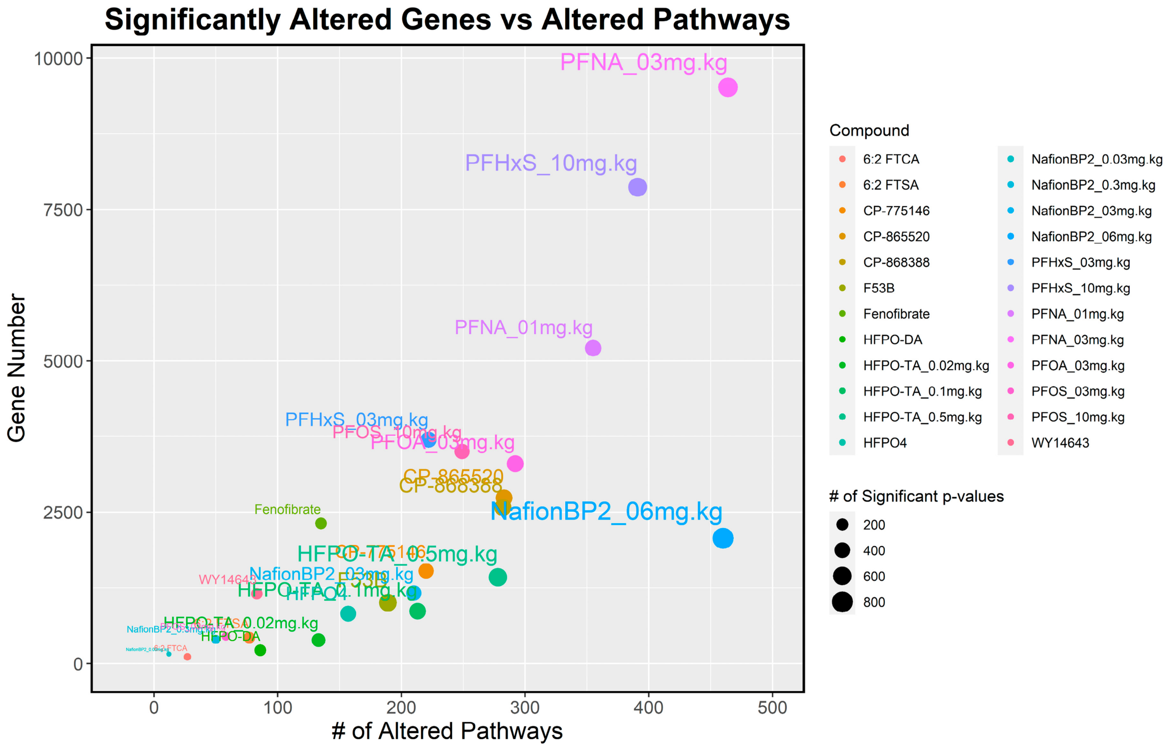

Fig S2B.

Figure S2: Relationship of number of altered genes, pathways, and their respective *p*-values from all datasets. (A) Bar plot of the total number of significant DEGs (± 1.2 fold change; *p*-value < 0.05) for each treatment group. Red and blue bars indicate the number of up- and down-regulated DEGs, respectively. The number above each bar is the total number of DEGs per group. The color of the treatment group labels represents the class of the compound. (B) Relationships between the number of genes and transcription factors altered. Scatterplot containing each dataset where the y axis corresponds to the number of significantly altered genes (±1.2 fold change; $p < 0.05$), and the x-axis

corresponds to the number of significantly altered pathways (utilizing a ±2 z-score cutoff). The size of the bubble represents the number of significantly altered pathways (p-value < 0.05) and the color represents each compound.

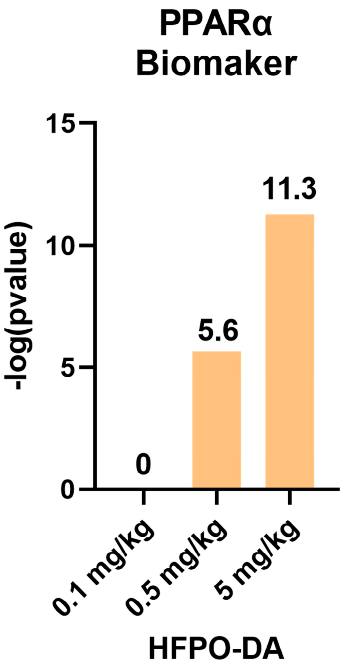

Figure S3: HFPO-DA induces PPARα activity in mice after a 90-day exposure. The dataset GSE135943 was analyzed through BSCE. In this study, three dose levels of HFPO-DA were administered to male mice by daily gavage for 90 days. RNA-Sequencing was performed on formalin fixed paraffin embedded liver tissues as described in the original study [49]. Each DEG list was compared to the PPARα biomarker in BSCE using the Running Fisher test. The p-values were exported and −log transformed. A bar plot is shown of the −log(p-value)s with the doses of HFPO-DA on the x-axis. The number above the bar is the −log(p-value).
